# Comparison of flexible ureteroscopic suction techniques: efficacy and safety of flexible and navigable access sheath (FANS) vs. direct in-scope suction (DISS) in the management of 2–3 cm lower pole renal stones

**DOI:** 10.1007/s00240-025-01748-7

**Published:** 2025-04-19

**Authors:** Ümit Yildirim, Mehmet Uslu, Mehmet Ezer, Çağlayan Erdoğdu, Ramazan Kocaaslan, Kemal Sarica

**Affiliations:** 1 Department of Urology, Faculty of Medicine, Kafkas University, Kars, Türkiye; 2 Department of Urology, Sancaktepe Şehit Prof.Dr. İlhan Varank Training And Research Hospital, Istanbul, Türkiye; 3 Department of Urology, Faculty of Medicine, Biruni University, Istanbul, Türkiye

**Keywords:** Flexible, Suction, Lower pole, Direct in scope, Navigable, Access sheath

## Abstract

This study compares the efficacy and safety of two techniques for flexible ureteroscopy (FURS) in managing 2–3 cm lower pole renal stones: the use of flexible and navigable access sheath (FANS) versus direct in-scope suction (DISS) without an access sheath. A retrospective analysis of 60 patients undergoing FURS for lower pole renal stones was conducted between March 2023 and January 2025. Group 1 (*n* = 32) underwent FANS-assisted procedures, while Group 2 (*n* = 28) underwent sheathless FURS with DISS. Stone-free rates (SFR) were assessed with non-contrast computed tomography (NCCT) after four weeks. Operative time, peri- and postoperative complications, and hospitalization duration were also compared. The mean operative time was significantly shorter in the FANS group (71.8 ± 11.5 min) compared to the DISS group (79.1 ± 11.7 min, *p* = 0.026). The difference between the SFRs obtained in the FANS group (62.5%) and the DISS group (46.4%) after the first session was not statistically significant (*p* = 0.162). After the second session, SFRs were also comparable (87.5% vs. 82.1%, *p* = 0.412) (Residual stones < 4 mm were considered stone-free). Completely stone-free rates (CSFR), defined as the absence of residual fragments after the first session were 43.7% in the FANS group and 32.1% in the DISS group (*p* = 0.256), while after the second session, these rates increased to 84.3% and 75%, respectively (*p* = 0.280). Postoperative fever rates (9.3% vs. 10.7%, *p* = 0.598) and hospitalization duration (2.5 ± 1.3 vs. 2.6 ± 1.4 days, *p* = 0.819) were similar. Both techniques achieved high overall SFRs for large lower pole stones in a staged manner. However, FANS was associated with a shorter operative time. While DISS eliminates the need for an access sheath, its longer operative time may be a limitation. Larger studies are needed to further evaluate these techniques.

## Introduction

The management of lower pole renal calculi presents unique challenges due to the anatomical characteristics hindering spontaneous passage and making endourological procedures in a more technically demanding position [1]. The European Association of Urology (EAU) guidelines recommend percutaneous nephrolithotomy (PCNL) as the gold standard treatment modality for stones larger than 2 cm due to its superior stone-free rates [2]. However, PCNL is associated with higher morbidity, including risks of bleeding, infection, and renal parenchymal injury, making alternative minimally invasive approaches advantageous, particularly for intermediate-sized stones measuring 2–3 cm [3, 4].

Flexible ureteroscopy (FURS) has emerged as a viable alternative for 2–3 cm renal stones, particularly with the advent of new game-changing technologies that allow suctioning during the procedure [5]. The most important examples of these newly introduced technologies are the flexible and navigable access sheaths (FANS), which gained wide acceptance recently, and disposable flexible ureterorenoscopes with direct in-scope suction (DISS). While FANS facilitate improved maneuverability, irrigation control, and fragment removal, DISS offers enhanced fragment evacuation, potentially reducing intrarenal pressure and minimizing the risk of infectious complications [6]. These innovations aim to improve the efficiency of stone disintegration and more importantly clearance by reducing the operative time as well as the need for staged procedures [7]. However, the necessity of an access sheath in optimizing these outcomes remains a subject of debate [8].

Considering the potential benefits of these techniques, it is essential to determine whether FURS with DISS, even without an access sheath, can achieve comparable outcomes to FANS-assisted procedures. Although a sufficient single-session stone-free rate may not yet be achievable in 2–3 cm renal stones [9], investigating whether these techniques can serve more effectively in selected cases remains an important clinical question.

This study aims at a comparative evaluation of the efficacy and safety of as well as the potential roles of FURS with FANS versus DISS without access sheath techniques in the minimally invasive management of 2–3 cm lower pole stones.

## Materials and methods

Between March 2023 and January 2025, departmental records of patients undergoing FURS procedures for large ( 2–3 cm) lower caliceal stones in our clinic were retrospectively reviewed. All ethical guidelines outlined in the Declaration of Helsinki were adhered to, and the institute’s ethics committee approved the study protocol (80576354-050-99 L/587). While all patients undergoing FURS for lower caliceal stones measuring between 2 and 3 cm were included, patients with ureteral stenosis, anatomical anomalies, ectopic kidney, solitary kidney, and previously inserted ureteral stents were excluded from the trial program. Included cases were divided into two groups according to the method used during FURS procedures as follows: Group FANS (*n* = 32) and Group DISS (*n* = 28).

Preoperative imaging assessments included non-contrast computed tomography (NCCT) and kidney-ureter-bladder (KUB) radiography to examine the anatomical structure of the lower calyceal system and main stone-related parameters. When necessary, urinary ultrasonography was performed for additional diagnostic assessment. Stone dimensions were determined based on the largest diameter outlined in NCCT sections. Prior to surgery, all patients underwent a urine culture test to confirm the absence of infection. In cases where urinary tract infections were detected, appropriate antibiotic therapy was administered. A single prophylactic dose of a second-generation cephalosporin was administered preoperatively. To assess the treatment’s effectiveness in terms of stone-free status, follow-up imaging with NCCT was conducted one month following surgery, both after the first and second sessions. Patients were classified as stone-free if no residual fragments were detected or if the remaining fragments were sizing ≤ 4 mm in size. The condition in which no remaining fragments were detected after the procedure was classified as completely stone-free and analyzed independently.

All procedures were performed by a surgeon with over 10 years of experience in endourology, under general anesthesia, with the patient in the dorsal lithotomy position. With the aid of a 9.5 Fr semi-rigid ureteroscope and a 0.035 Fr guidewire, access to the renal pelvis was successfully performed under fluoroscopic guidance. A detailed evaluation of the pelvicalyceal anatomy was then performed using retrograde pyelography, allowing for a comprehensive assessment of the collecting system characteristics.

**Fig. 1:**
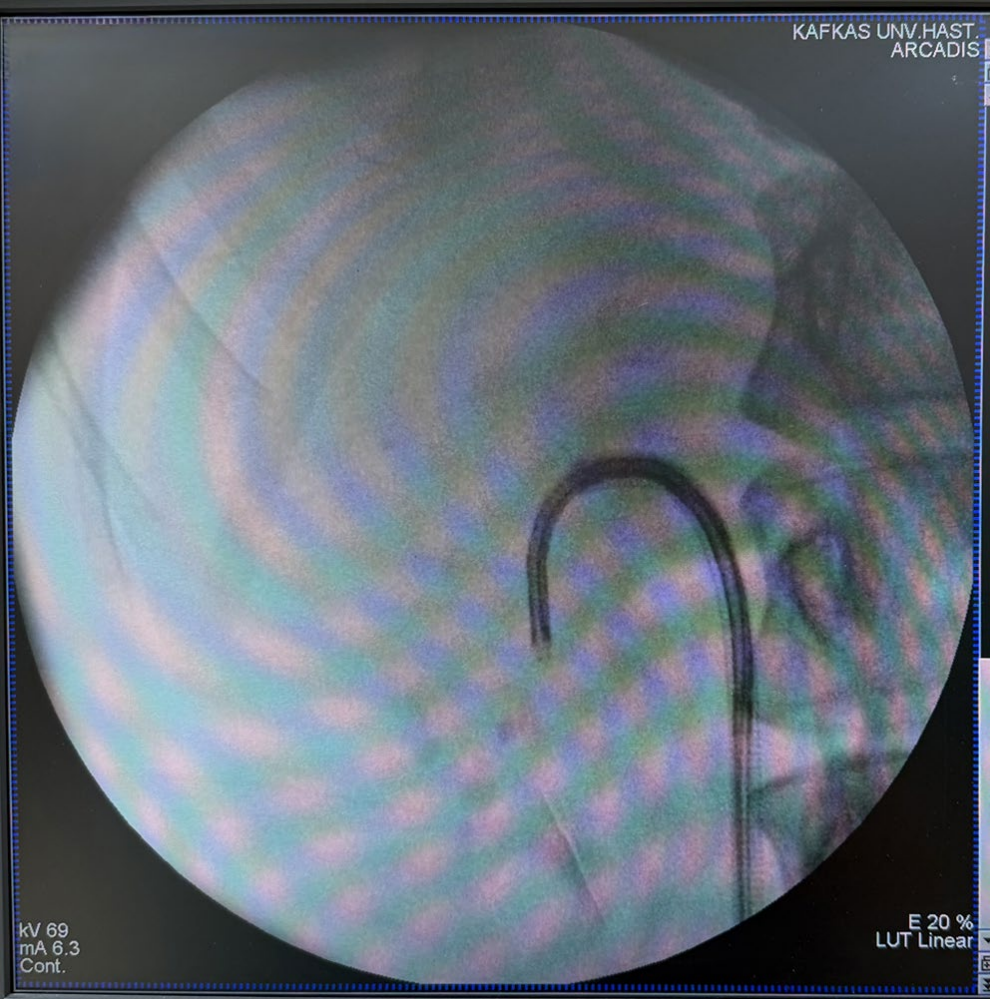
Flexible and navigable access sheath that pushed into the lower renal calyceal position under the guidance of the flexible ureterorenoscope

After this stage, in patients undergoing the FANS procedure an access sheath (12/14 Fr) (ClearPetra; Well Lead Medical, Guangzhou, China) with a bendable tip and aspiration feature was placed into the relevant ureter up to the renal pelvis over the guidewire. The single-use flexible scope (7.5 Fr) ( HugeMed Medical Technical Development Co.; Shenzhen, China) was passed through the access sheath, and the renal collecting system was examined in detail. After this maneuver, the tip of the placed access sheath was pushed into the lower renal calyceal position under the guidance of the flexible URS (Fig. 1), and a holmium fiber (Litho 35 W Holmium laser, Quanta System, Samarate, Italy) was used to pulverize the stones with a 273-µm fiber. In this group, stones were disintegrated ( fragmented) into an adequate size (< 3 mm) to allow an efficient extraction with the help of active aspiration.

**Fig. 2:**
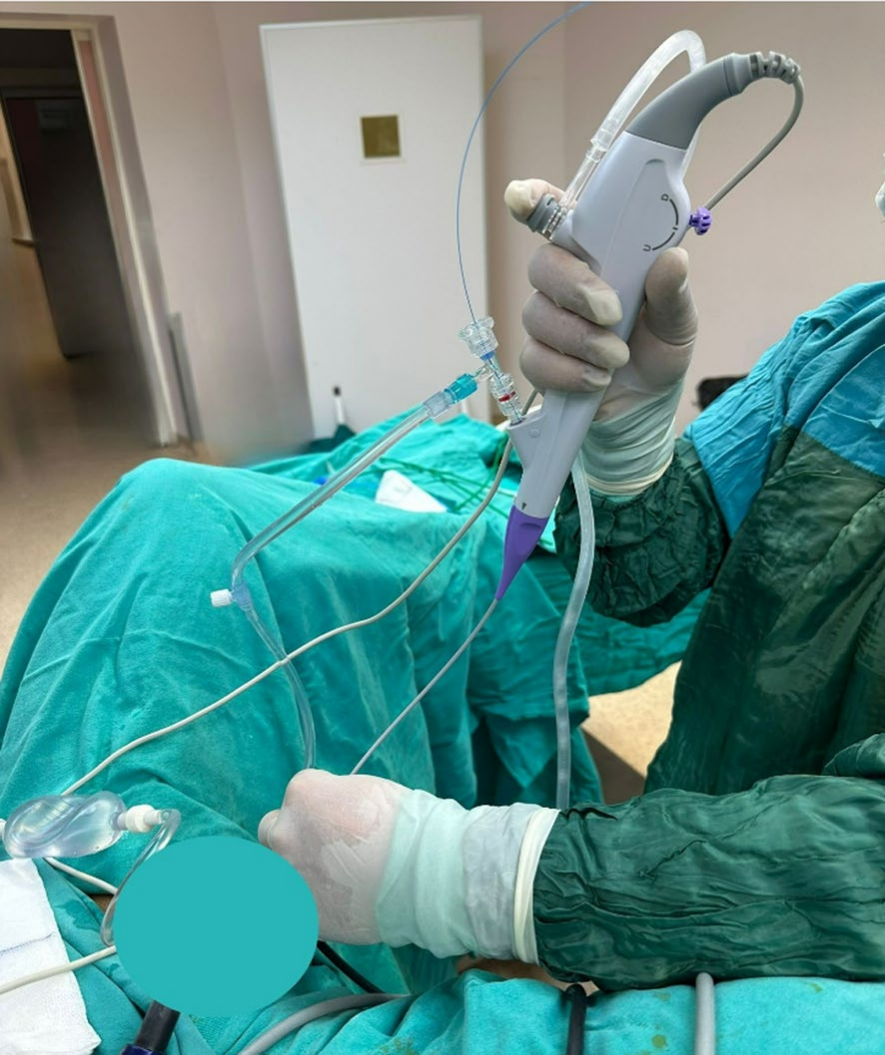
Direct in-scope suction system that introduced over the placed guidewire and navigated into the renal pelvis without the use of an access sheath

In the DISS group, a single-use flexible URS (8.4 Fr) ( Scivita Medical Technology Co.; Jiangsu, China) with a direct in-scope suction system was introduced over the placed guidewire and navigated into the renal pelvis without the use of an access sheath (Fig. 2) (Figures were taken from our institutional archive). The suctioning port on the 8.4 Fr ureteroscope is 3.6 Fr and the laser fiber operates via the same port. In this group, the dusting preset of the same laser device was used to disintegrate the stones. While continuous irrigation was applied to ensure optimum visualization, the ventilation slot on the ureterorenoscope was used by the operator when deemed necessary to prevent excessive increase in intrarenal pressure. In addition, dust formations were eliminated by the vacuum effect.

In both groups, a 4.8 Fr double-J stent was placed in all patients after the first and second session surgeries.

Statistical analyses were conducted using SPSS version 22.0 (SPSS Inc., Chicago, IL, USA). Continuous variables were presented as means with standard deviations (SD) for normally distributed data, whereas non-normally distributed variables were expressed as medians with interquartile ranges (IQR). Group comparisons for continuous variables were performed using the independent samples t-test for normally distributed data and the Mann-Whitney U test for non-normally distributed data. Categorical variables were reported as frequencies and percentages and were analyzed using the Chi-square test or Fisher’s exact test, as appropriate.

## Results

Baseline characteristics, including age, gender distribution, and all patient and stone-related parameters were comparable between the two groups, with no statistically significant differences. The baseline patient characteristics and clinical findings are given in Table 1.

**Table 1:** Patients’ characteristics and clinical findings

		Group FANS (*n* = 32)		Group DISS (*n* = 28)		*p*
Gender^a^	Male	23	71.8%	19	67.8%	0.477
	Female	9	28.1%	9	32.1%	
Age^b^		51.6	± 13.1	48.8	± 14.5	0.419
Diabetes^a^		3	9.3%	3	10.7%	0.435
Charlson Comorbidity Index^c^		1	[1–3]	1	[0–3]	0.350
Body Mass Index (kg/m²)^b^		28.6	± 3.6	26.6	± 3.5	0.377
Preoperative Hemoglobin (g/dL)^b^		14.7	± 1.7	14.2	± 1.5	0.382
Preoperative Creatinine (mg/dL)^b^		1.0	± 0.3	0.9	± 0.3	0.293
Lateralization^a^	Right	13	40.6%	13	46.4%	0.229
	Left	19	59.3%	15	53.5%	
Stone Size (mm)^b^		25.6	± 2.4	24.7	± 3.0	0.166
Stone Density (HU)^b^		879.1	± 294.2	917.8	± 289.6	0.553
Opacity^a^	Opaque	26	81.2%	23	82.1%	0.598
	Non-opaque	6	18.7%	5	17.8%	
Hydronephrosis^a^		15	46.8%	12	42.8%	0.480
Stone Number^a^	Single	10	31.2%	11	39.2%	0.352
	Multiple	22	68.7%	17	60.7%	
Previous Endoscopic Stone Surgery^a^		19	59.3%	17	60.7%	0.563
Parenchymal Thickness (mm)^b^		21.7	± 4.8	22.3	± 4.9	0.660
Infundibolopelvic Angle (°)^b^		63.4	± 13.5	65.1	± 8.3	0.303

^a^:data are presented as count and percentage, ^b^: data are given as mean ± standard deviation, ^c^: data are presented as median (IQR)

As an important operative parameter that may affect the course of the procedure, the mean operative time was significantly shorter in the FANS group (71.8 ± 11.5 min) compared to the DISS group (79.1 ± 11.7 min, *p* = 0.026). Likewise, postoperative hemoglobin and creatinine levels remained comparable in both groups, with no significant differences in postoperative biochemical outcomes.

Regarding the stone-free rates (SFR), while 62.5% of patients in the FANS group achieved stone clearance after the first session, this value was 46.4% in the DISS group (*p* = 0.162). After a second session, the overall SFR increased to 87.5% in the FANS group and 82.1% in the DISS group (*p* = 0.412). When considering the completely stone-free rate (CSFR) (absence of any residual fragments), 43.7% of patients in the FANS group achieved complete clearance after the first session, compared to 32.1% in the DISS group (*p* = 0.256). Following the second session, the completely stone-free rate increased to 84.3% in the FANS group and 75% in the DISS group (*p* = 0.280).

Regarding complications, postoperative fever was observed in 9.3% of FANS patients and 10.7% of DISS patients (*p* = 0.598) after the first session. The fever remained below 38 °C, and all patients recovered within 24 h with supportive care. No cases experienced sepsis in either group of cases. Perirenal hematoma due to renal cyst rupture was observed in 1 patient in the FANS group. It regressed during 1 week of follow-up without any specific approach. There was no record of postoperative fever or any other complications after the second session of interventions. A summary of postoperative follow-up data is given in Table 2.

**Table 2:** Postoperative follow-up data

	Group FANS (*N* = 32)		Group DISS (*N* = 28)		*p*
Postoperative Hemoglobin(g/dL)^b^	14.0	± 1.7	14.1	± 1.4	0.906
Postoperatieve Creatinine (mg/dL)^b^	0.9	± 0.2	0.9	± 0.3	0.472
SFR After First Session^a^	20	62.5%	13	46.4%	0.162
Completely SFR After First Session^a^	14	43.7%	9	32.1%	0.256
SFR After Second Session^a^	28	87.5%	23	82.1%	0.412
Completely SFR After Second Session^a^	27	84.3%	21	75%	0.280
Postoperative Fever^a^	3	9.3%	3	10.7%	0.598
First Session Operative Time (minutes)^b^	71.8	± 11.5	79.1	± 11.7	0.026
Largest Daimeter of Residual Stones Before Second Session (mm)	7.9	± 1.4	8.4	± 1.9	0.410
Second Session Operative Time (minutes)^b^	53.6	± 6.2	68.5	± 6.9	< 0.001
Hospitalization Duration (days)^b^	2.5	± 1.3	2.6	± 1.4	0.819

SFR: stone-free rate. ^a^: data are presented as count and percentage, ^b^: data are given as mean ± standard deviation

## Discussion

Our study aimed to compare the efficacy and safety of the FURS procedure performed with two different techniques namely FANS versus DISS in the management of large ( 2–3 cm) lower pole renal stones. While the SFR after the first session was lower in the DISS group, the overall SFR after two sessions was comparable and exceeded 80% in both groups. This finding clearly demonstrates that both methods can achieve high success rates with additional sessions. However, despite comparable success rates, our findings showed a longer operation time against DISS, a critical disadvantage that can be attributed to the dusting effect used in stone pulverization, which definitely requires a longer time for effective stone evacuation. Previous studies have demonstrated that mini-PCNL offers superior single-session SFRs for renal stones in the 2–3 cm range [10]. However, PCNL techniques are associated with higher complication rates, which may sometimes be severe enough to include bleeding, infection, and renal parenchymal injury [5]. On the other hand, there are also studies suggesting that there is no significant difference in complications between the two procedures when mini-PCNL and FURS are compared for the treatment of lower calyceal stones [11]. Our current results demonstrate that as acceptable alternative options, both FANS and DISS could provide efficient and safe outcomes in the minimally invasive management of 2–3 lower calyceal stones in a staged manner. Although not statistically significant, the slightly higher single-session SFR observed with FANS may be attributed to its enhanced fragment evacuation via direct suctioning, potentially minimizing the need for prolonged dusting and irrigation.

Several previous studies have assessed the efficacy of FANS and DISS in endoscopic stone management. According to some of these studies, FANS in flexible ureteroscopy resulted in a significantly shorter operative time and improved fragment retrieval efficiency compared to the use of conventional access sheats [12]. Similarly, outcomes of some studies revealed that DISS reduced intrarenal pressure and facilitated better visualization, although prolonged operational time [13, 14]. These findings align well with our current results, highlighting the trade-off between better evacuation efficiency in FANS and prolonged operative times in DISS. When interpreting these findings, it should be kept in mind that as the main advantage of the technique we applied; patients in the DISS group did not require the use of an extra access sheath. Given the well-documented risks associated with access sheaths, their avoidance in this group may be beneficial [15].

Additionally, recent reviews have suggested that suction techniques offer slightly higher SFRs in the first session, minimizing the risk of infectious complications [16]. This aligns with our observation that postoperative fever rates were comparable between the patients of both groups, despite the different suction mechanisms employed.

Moreover, recent advancements in laser technology, particularly thulium fiber laser (TFL), may further enhance stone pulverization efficiency and reduce operative time. TFL has been shown to provide superior fragmentation rates with reduced retropulsion, potentially increasing SFR and shortening procedure duration compared to conventional 35 W holmium: YAG lasers [17]. TFL technology, in combination with smaller laser fibers and advanced accessories, has significantly improved the effectiveness and safety of lower pole stone management. The combination of TFL with different suction devices is increasingly reported as a promising method that can greatly enhance stone-free rates (SFR) by facilitating better fragment clearance and minimizing intrarenal pressure [18]. It was not difficult to predict that in future studies, as a less invasive alternative than PNL approaches, a combination of FANS and DISS methods with TFL could achieve higher SFR ​​in a single session. In the treatment of medium-sized lower calyceal stones.

To summarize, when comparing our findings with the data reported so far on PCNL approaches in the literature so far, it is important to note that PCNL generally achieves single-session SFRs exceeding 90% for similar stone size and location [19]. However, this benefit comes at the expense of significantly higher morbidity, including an increased risk of severe complications such as sepsis, hemorrhage, and prolonged hospitalization [20]. In light of these facts, our study underscores the viability of FURS-based approaches as less invasive alternatives, particularly for patients who prioritize a reduced risk profile over immediate single-session stone clearance.

### Limitations

One potential limitation of our study is the lack of a PCNL or mini-PCNL control group. However, it is crucial to emphasize that our objective was not to compare these already well-established techniques but rather to determine the efficacy of FANS and DISS techniques in a comparative manner The efficacy of PCNL and mini-PCNL for renal stones in this size range has been extensively validated in prior studies, and thus, their omission should not be considered a major limitation of our analysis.

Another limitation is the retrospective nature of our study, which may introduce selection bias. Additionally, the relatively small sample size could have negatively affected the statistical power of our findings. A larger cohort might have provided more definitive conclusions regarding the comparative efficacy of FANS and DISS. However, taking the highly limited data reported so far on this issue in the literature, we believe that our current results will be contributive enough to the published information. Future prospective studies with a larger number of participants are necessary to validate our results and strengthen the statistical significance of our observations.

## Conclusion

Our study provides valuable insights into the comparative evaluation of the safety and efficacy of FANS vs. DISS applications in the treatment of 2–3 cm lower pole renal stones. Although the first-session SFR was lower in the DISS group, both techniques ultimately achieved high overall SFRs after the second session. The prolonged operative time in the DISS group highlights a potential drawback of this technique, likely due to the dusting effect required for stone disintegration. Given the low complication rates, particularly in patients favoring a minimally invasive approach, these techniques represent a valuable alternative for managing such stones in selected cases. Future prospective studies with larger cohorts are warranted to refine treatment strategies and further optimize patient outcomes.

## Data Availability

No datasets were generated or analysed during the current study.
